# Large-Scale Genetic Correlation Analysis between Spondyloarthritis and Human Blood Metabolites

**DOI:** 10.3390/jcm12031201

**Published:** 2023-02-02

**Authors:** Mingyi Yang, Jiawen Xu, Feng Zhang, Pan Luo, Ke Xu, Ruoyang Feng, Peng Xu

**Affiliations:** 1 Department of Joint Surgery, HongHui Hospital, Xi’an Jiaotong University, Xi’an 710054, China; 2 Orthopedic Research Institute, Department of Orthopedics, West China Hospital, Sichuan University, Chengdu 610041, China; 3 Key Laboratory of Trace Elements and Endemic Diseases, National Health and Family Planning Commission, School of Public Health, Health Science Center, Xi’an Jiaotong University, Xi’an 710061, China

**Keywords:** spine, joints, blood, genetic, metabolite

## Abstract

The aim was to study the genetic correlation and causal relationship between spondyloarthritis (SpA) and blood metabolites based on the large-scale genome-wide association study (GWAS) summary data. The GWAS summary data (3966 SpA and 448,298 control cases) of SpA were from the UK Biobank, and the GWAS summary data (486 blood metabolites) of human blood metabolites were from a published study. First, the genetic correlation between SpA and blood metabolites was analyzed by linkage disequilibrium score (LDSC) regression. Next, we used Mendelian randomization (MR) analysis to perform access causal relationship between SpA and blood metabolites. Random effects inverse variance weighted (IVW) was the main analysis method, and the MR Egger, weighted median, simple mode, and weighted mode were supplementary methods. The MR analysis results were dominated by the random effects IVW. The Cochran’s Q statistic (MR-IVW) and Rucker’s Q statistic (MR Egger) were used to check heterogeneity. MR Egger and MR pleiotropy residual sum and outlier (MR-PRESSO) were used to check horizontal pleiotropy. The MR-PRESSO was also used to check outliers. The “leave-one-out” analysis was used to assess whether the MR analysis results were affected by a single SNP and thus test the robustness of the MR results. Finally, we identified seven blood metabolites that are genetically related to SpA: X-10395 (correlation coefficient = −0.546, *p* = 0.025), pantothenate (correlation coefficient = −0.565, *p* = 0.038), caprylate (correlation coefficient = −0.333, *p* = 0.037), pelargonate (correlation coefficient = −0.339, *p* = 0.047), X-11317 (correlation coefficient = −0.350, *p* = 0.038), X-12510 (correlation coefficient = −0.399, *p* = 0.034), and X-13859 (Correlation coefficient = −0.458, *p* = 0.015). Among them, X-10395 had a positive genetic causal relationship with SpA (*p* = 0.014, OR = 1.011). The blood metabolites that have genetic correlation and causal relationship with SpA found in this study provide a new idea for the study of the pathogenesis of SpA and the determination of diagnostic indicators.

## 1. Introduction

Spondyloarthritis (SpA) is a group of phenotypically different but related diseases, including psoriatic arthritis (PsA), arthritis associated with inflammatory bowel disease, reactive arthritis, juvenile idiopathic arthritis (JIA), and ankylosing spondylitis (AS) [1]. SpA is divided into the following two categories: axial and peripheral [2]. SpA is an autoinflammatory disease, not an autoimmune disease driven by T cells or B cells. The main feature of SpA is the variability of its clinical manifestations that are often accompanied by bone inflammation and extra-articular features. Bone destruction and bone erosion are prominent features of SpA [3]. The clinical forms of SpA include spinal (axial) and extra-articular features along with peripheral arthritis [1]. The incidence of SpA is 0.48~63/100,000, and the prevalence is 0.01~2.5% [2]. The prevalence of SpA in adult Caucasians is 0.3%, and it is the second most common subtype of chronic inflammatory rheumatic diseases, second only to rheumatoid arthritis (RA) [4]. The prevalence of SpA in the adult population is approximately 1.73% [5]. The prevalence of SpA in most parts of Asia is lower than that of other countries in the world. The prevalence of SpA in China is 0.49~0.93% [6]. SpA is a serious threat to human health, but its pathogenesis is still unclear.

SpA is also associated with genetics and the environment. The major genetic risk factor for SpA is human leucocyte antigen-B27 (HLA-B27), which is a major histocompatibility complex class 1 antigen (MHC I). There is a degree of association between different HLA-B27 subtypes and AS [7]. The recurrence rate of AS in the first-degree relatives of SpA patients is approximately 12%, and strong genetic susceptibility is also applicable to other types of SpA [1]. The genes encoding the HLA-B27 and interleukin-23 (IL-23) receptors are related to different SpA subtypes. There is a dominant shared genetic factor across all types of SpA, and other genetic factors and environmental factors contribute to phenotypic diversity [8]. All phenotypes of familial SpA seem to be related to the same factors, and the phenotypic variation of these factors may be derived from the same disease, including axial disease and the two main subtypes of more diffuse joint and extra-articular diseases [9]. The study also showed that in multiple-case families, phenotypic variations corresponding to the SpA subtype were derived from the same disease [8,10,11]. Studies have found that HLA-B27 transgenic rats can develop not only SpA but also peripheral arthritis, colitis, uveitis, and skin diseases. Environmental factors (such as gut microbiota) and other genetic factors determine the exact phenotype [12,13]. The genome-wide association study (GWAS) found that endoplasmic reticulum aminopeptidase 1 (ERAP1), ERAP2, and IL-23R are susceptibility factors for SpA in addition to HLA-B27 and that the IL-23/IL-17 pathway is very important in the SpA pathogenesis [14,15,16].

The metabolome is a collection of cellular metabolites, the small molecules that participate in cell metabolism [17]. Sex, age, physiology, behavior, and lifestyle (such as diet) may cause changes in metabolite levels. Genetic differences not only directly cause metabonomics variation but also may influence physiology, behavior, and lifestyle at the genetic level [18]. Common genetic variations in human metabolism have been analyzed through genomics and metabolomics, and metabolites affected by genetics have been identified [19]. At present, GWAS has found correlations between hundreds of single-nucleotide polymorphisms (SNPs) and various metabolite categories in different populations [19]. Studies have estimated the heritability of 361 blood metabolites in 40 GWAS [18]. A study examining endogenous peptides and metabolites in serum of patients with AS found that vitamin D3 metabolite-(23S,25R)-25-hydroxyvitamin D3 26,23-peroxylactone was down-regulated in AS [20]. A metabolomic fingerprinting study found that the selected long-chain fatty acids (e.g., 3-hydroxytetradecanedioic acid) that are associated with dysregulation of fatty acid metabolism were up-regulated in patients with severe PsA. Furthermore, a number of different eicosanoids with either pro- or anti-inflammatory properties were detected solely in serum samples of patients with moderate and severe PsA [21]. A metabolomic study of serum samples found greater changes in serum metabolites in patients with AS compared to patients with PsA [22]. In addition, a study exploring the potential of serum metabolic analysis in SpA patients showed great potential for the diagnosis of SpA, prediction of peripheral disease, and assessment of disease activity and response to treatment [23]. However, the genetic correlation between SpA and blood metabolites needs to be further explored. A better understanding of the genetic correlation between SpA and blood metabolites will help us understand the cause of the disease.

Genetic correlation is a major parameter used to describe the genetic correlation between traits and traits or between traits and diseases. Linkage disequilibrium score (LDSC) regression is one of the most commonly used methods to analysis the genetic correlation [24]. Confounding factor bias (obscure genetic relationship and population stratification) and polygenic effects in GWAS were the main reasons for the expansion of test statistics. LDSC can correct the polygenic effects in the GWAS analysis. The contribution of each variable can be quantified by introducing the regression relationship between the LDSC detection statistics and LD (r2), and the intercept value of the regression analysis can also be used to distinguish the cause of statistical expansion and correct it [25,26]. Mendelian randomization (MR) uses genetic variation as an instrumental variable (IVs) to assess genetic causality between risk factors and outcomes [27]. MR follows the Mendelian rule that “parental alleles are randomly assigned to offspring”. If the genotype determines the phenotype, then the genotype is related to the disease through the phenotype, so the genotype can be used as an IVs to infer the correlation between phenotype and disease [26].

In this study, LDSC and MR were used to comprehensively evaluate the genetic relationship between SpA and human blood metabolites. The analysis of these molecular genetic data can not only evaluate the common inheritance patterns between SpA and blood metabolites but also evaluate the potential genetic causal relationship between SpA and blood metabolites. The findings of this study strengthen our understanding of the genetic mechanism of SpA and indicate new directions for identifying diagnostic indicators of SpA.

## 2. Materials and Methods

### 2.1. The GWAS Summary Data of SpA

The GWAS summary data of SpA came from the UK Biobank. In short, a total of 462,933 samples of males and females of European descent from the UK Biobank were included, including 4033 SpA patients and 458,900 controls, with 9,851,867 SNPs analyzed. All subjects obtained ethical approval and signed informed consent. The Affymetrix UK BiLEVE Axiom or UK Biobank Axiom array was applied to genotype the UK Biobank participants. The interpolation of 90 million genetic variants from the Haplotype Reference Consortium, the 1000 Genomes Project, and the UK 10K project supplemented the data [28,29]. After quality control, the last study group included 452,264 samples, including 3966 SpA and 448,298 controls. Comprehensive information on the samples, genotyping, augmentation, and quality control has previously been published [30].

### 2.2. The GWAS Summary Data of Human Blood Metabolite

The GWAS summary data of human blood metabolite came from a published study [31]. In short, the study measured 529 metabolites in the plasma or serum of 7824 adults of the two European population studies, the TwinsUK cohort and the Cooperative Health Research in the Region of Augsburg (KORA) project. All subjects obtained ethical approval and signed informed consent. Liquid-phase chromatography and gas chromatography separation coupled with tandem mass spectrometry was used for metabolite analysis. MERLIN software was used for association analysis in the TwinsUK study. QUICKTEST software was used to fit a linear model in the KORA study. Approximately 2.1 million SNPs were genotyped. HapMap2 and 1000 Genomes Project (1000 Genomes Project, 1KGP) data were used to re-estimate the association of each site to investigate the genetic variation of metabolic sites more comprehensively. The 486 metabolites in the two cohorts could be used for genetic analysis after strict quality control. Detailed information about the data, genotyping, estimation, and association analysis has been published previously [31].

### 2.3. LDSC Regression

LDSC is a newly developed SNP heritability estimation method that can estimate genetic correlation through GWAS summary data instead of a single level of genotype data [32,33]. The data required for LDSC are the outcome of GWAS, the explanatory variables in the regression are the LD score of SNP sites, and the dependent variables are the core of the algorithm. To evaluate if there were confounding factors in the GWAS results, a custom statistic having to conform to the chi-square distribution was defined, and the connection between the LD score and the chi-square statistic was equipped using linear regression [34]. The principle of LDSC is that genetic variants present in more genomes are more likely to be causal variants than genetic variants with low LD scores, so the average statistics of genetic variants with high LD scores are higher [35]. Polygenicity as well as confounding biases such as cryptic relatedness and species stratified can result in an enlarged distribution of test statistics in GWAS. The LDSC quantifies the contribution of each variable by examining the relationship between the test statistic and LD [34]. We used the LDSC analysis software (https://github.com/bulik/ldsc, accessed on 22 February 2021) to analysis the genetic correlation between SpA and blood metabolite.

### 2.4. MR Analysis

The blood metabolites that had genetic correlation with SpA obtained by LDSC regression were analyzed by MR. MR analysis requires satisfaction with three basic assumptions: (a) IVs were correlated with exposure; (b) IVs were not associated with confounding factors between exposure and outcome; and (c) IVs affected outcome only through exposure.

In order to ensure the robustness of MR analysis results, IVs were strictly screened. First, we obtained SNPs associated with blood metabolites (*p* < 5 × 10^−6^) [36]. Second, to eliminate the LD between the included IVs, the clumping process (r2 < 0.001, clumping distance = 10,000 kb) was used. Third, we excluded SNPs linked to SpA (*p* < 5 × 10^−6^). Fourth, we used the PhenoScanner database (http://www.phenos-canner.medschl.cam.ac.uk/phenoscanner, accessed on 12 December 2022) to rule out SNPs related to confounding factors [37]. Previous literature has shown that the main risk factors for SpA are age and smoking [38]. Fifth, palindromic SNPS were not included in our study.

Random effects inverse variance weighted (IVW) was the main analysis method, and the MR Egger, weighted median, simple mode, and weighted mode were supplementary methods. Our analysis results mainly refer to the random effects IVW method. The Cochran’s Q statistic (MR-IVW) and Rucker’s Q statistic (MR Egger) were used to check heterogeneity, and *p* > 0.05 was considered to have no heterogeneity [39]. MR Egger and MR pleiotropy residual sum and outlier (MR-PRESSO) method were used to detect whether there was horizontal pleiotropy in our MR analysis. The intercept test (MR Egger) and global test (MR-PRESSO) were used to check horizontal pleiotropy, and *p* > 0.05 was considered to have no horizontal pleiotropy [37]. Further, the distortion test (MR-PRESSO) was used to check outliers [34]. If the MR analysis results were affected by outliers, we re-evaluated the causal relationship after removing the outliers. Finally, the “leave-one-out” analysis was used to assess whether the genetic assessment results were affected by a single SNP and thus test the robustness of the MR results [40]. If the MR analysis results were affected by a single SNP, in order to avoid the occurrence of false positives or false negatives to the maximum extent, we re-evaluated the causal relationship after removing the single SNP that affected the MR analysis results.

## 3. Results

### 3.1. Genetic Correlation Analysis

**Figure 1:**
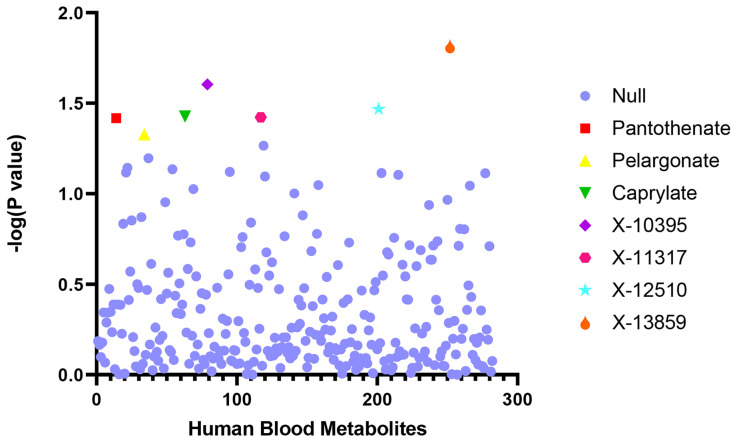
Genetic correlation analysis between spondyloarthritis and blood metabolites. Abscissa: the number of blood metabolites. Ordinate: *p*-value of the linkage disequilibrium score regression results. Null: The linkage disequilibrium score regression showed the blood metabolites that had no genetic correlation with spondyloarthritis (*p*-value > 0.05).

The LDSC software was used to analyze the genetic correlation between SpA and blood metabolites. The following seven blood metabolites genetically related to SpA were found: X-10395 (correlation coefficient = −0.546, *p* = 0.025), pantothenate (correlation coefficient = −0.565, *p* = 0.038), caprylate (correlation coefficient = −0.333, *p* = 0.037), pelargonate (correlation coefficient = −0.339, *p* = 0.047), X-11317 (correlation coefficient = −0.350, *p* = 0.038), X-12510 (correlation coefficient = −0.399, *p* = 0.034), and X-13859 (correlation coefficient = −0.458, *p* = 0.015) (Table 1, Figure 1).

### 3.2. Genetic Causal Analysis

After strict quality control, we obtained 20 IVs to perform the MR analysis of X-10395 and SpA, and there were no palindromic SNPs. We obtained 12 IVs to perform the MR analysis of pantothenate and SpA, and there was one palindromic SNP (rs248063). We obtained 29 IVs to perform the MR analysis of caprylate and SpA, and there was one palindromic SNP (rs7816456). We obtained 23 IVs to perform the MR analysis of pelargonate and SpA, and there were four palindromic SNPs (rs10882819, rs10967809, rs12704178, and rs2421124). We obtained 27 IVs to perform the MR analysis of X-11317 and SpA, and there was one palindromic SNP (rs10763882). We obtained 9 IVs to perform the MR analysis of X-12510 and SpA, and there were no palindromic SNPs. We obtained 29 IVs to perform the MR analysis of X-13859 and SpA, and there were 3 palindromic SNPs (rs10763882, rs12098046, and rs7253788). No IVs were associated with outcome and confounding factors (Supplementary Table S1).

**Figure 2:**
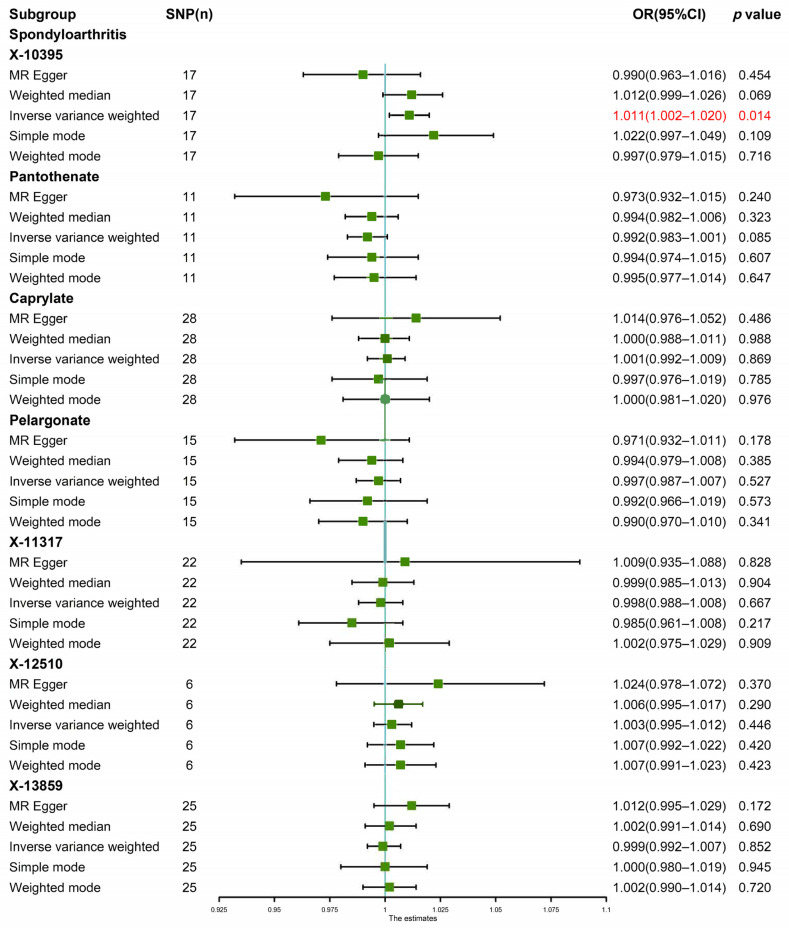
MR analysis results of the blood metabolites (X-10395, pantothenate, caprylate, pelargonate, X-11317, X-12510, and X-13859) and spondyloarthritis. Five methods: random-effects IVW, MR Egger, weighted median, simple mode, and weighted mode. The red part of the figure is the results that had genetic causal relationship.

**Figure 3:**
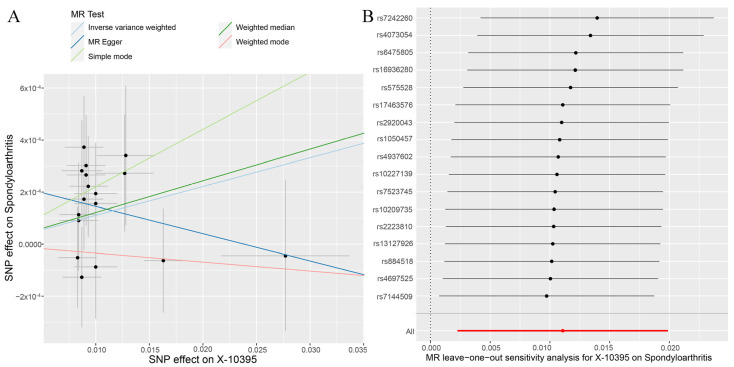
MR analysis results of the X-10395 and spondyloarthritis. (**A**) Scatter plot; (**B**) leave-one-out analysis.

The random effects IVW results showed that X-10395 had a positive genetic causal relationship with SpA (Supplementary Figure S1A,B). The Cochran’s Q statistic (MR-IVW) and Rucker’s Q statistic (MR Egger) showed that there was no heterogeneity (*p* > 0.05). The intercept test (MR Egger) and global test (MR-PRESSO) showed that there was no horizontal pleiotropy (*p* > 0.05). The distortion test (MR-PRESSO) showed that there were no outliers (Supplementary Table S2). The “leave-one-out” analysis showed that the MR results were affected by one SNP (rs7160179) (Supplementary Figure S1B). Therefore, the second round of MR analysis was performed after rs7160179 was removed. The random effects IVW results showed that X-10395 had no genetic causal relationship with SpA (Supplementary Figure S1C,D), and there were no instances of heterogeneity, horizontal pleiotropy, and outliers (Supplementary Table S2). However, the “leave-one-out” analysis showed that the MR results were affected by two SNPs (rs1800775 and rs5758064) (Supplementary Figure S1D). Finally, the third round of MR analysis was performed after rs1800775 and rs5758064 were removed. The random effects IVW results showed that X-10395 had a positive genetic causal relationship with SpA (*p* = 0.014, OR = 1.011). The MR Egger, weighted median, simple mode, and weighted mode showed that X-10395 had no genetic causal relationship with SpA (Figure 2 and Figure 3A). There were no instances of heterogeneity, horizontal pleiotropy, and outliers (Table 2), and the “leave-one-out” analysis showed that the MR results were not affected by a single SNP (Figure 3B).

**Figure 4:**
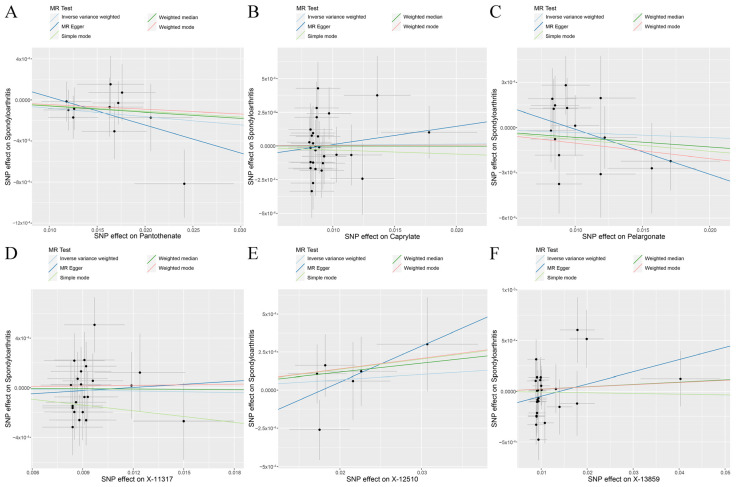
Scatter plot of MR analysis results between blood metabolites (pantothenate, caprylate, pelargonate, X-11317, X-12510, and X-13859) and spondyloarthritis. (**A**) Pantothenate and spondyloarthritis; (**B**) caprylate and spondyloarthritis; (**C**) pelargonate and spondyloarthritis; (**D**) X-11317 and spondyloarthritis; (**E**) X-12510 and spondyloarthritis; (**F**) X-13859 and spondyloarthritis.

**Figure 5:**
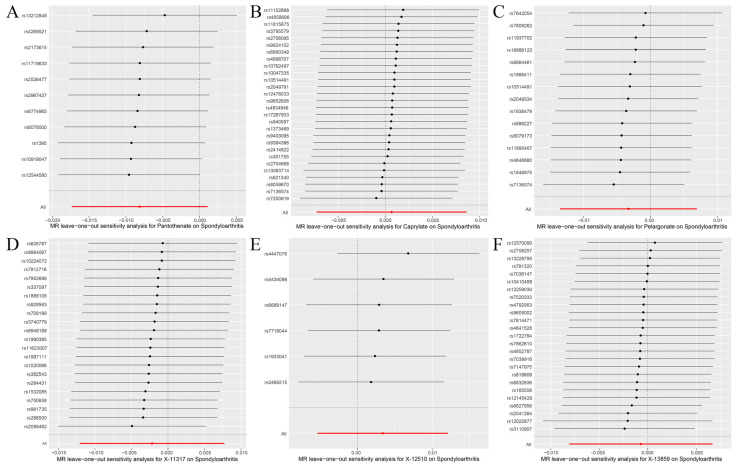
Leave-one-out analysis of MR analysis results between blood metabolites (pantothenate, caprylate, pelargonate, X-11317, X-12510, and X-13859) and spondyloarthritis. (**A**) Pantothenate and spondyloarthritis; (**B**) caprylate and spondyloarthritis; (**C**) pelargonate and spondyloarthritis; (**D**) X-11317 and spondyloarthritis; (**E**) X-12510 and spondyloarthritis; (**F**) X-13859 and spondyloarthritis.

The random effects IVW results showed that pantothenate (*p* = 0.085, OR = 0.992) and caprylate (*p* = 0.869, OR = 1.001) had no genetic causal relationship with SpA. The MR Egger, weighted median, simple mode, and weighted mode were consistent with the random effects IVW results (Figure 2 and Figure 4A,B). The Cochran’s Q statistic (MR-IVW) and Rucker’s Q statistic (MR Egger) showed that there was no heterogeneity (*p* > 0.05). The intercept test (MR Egger) and global test (MR-PRESSO) showed that there was no horizontal pleiotropy (*p* > 0.05). The distortion test (MR-PRESSO) showed that there were no outliers (Table 2). The “leave-one-out” analysis showed that the MR results were not affected by a single SNP (Figure 5A,B).

The random effects IVW results showed that pelargonate had no genetic causal relationship with SpA (Supplementary Figure S2A,B). The Cochran’s Q statistic (MR-IVW) and Rucker’s Q statistic (MR Egger) showed that there was heterogeneity (*p* < 0.05). The intercept test (MR Egger) showed that there was no horizontal pleiotropy (*p* > 0.05), but the global test (MR-PRESSO) showed that there was horizontal pleiotropy (*p* < 0.05). The distortion test (MR-PRESSO) showed that there were no outliers but showed four potential outliers (rs10848204, rs1447842, rs511545, and rs9033) (Supplementary Table S2). The “leave-one-out” analysis showed that the MR results were not affected by a single SNP (Supplementary Figure S2B). In order to remove horizontal pleiotropy, we performed a second round of MR analysis after removing the four potential outliers. The random effects IVW results showed that pelargonate had no genetic causal relationship with SpA (*p* = 0.527, OR = 0.997). The MR Egger, weighted median, simple mode, and weighted mode were consistent with the random effects IVW results (Figure 2 and Figure 4C). There were no instances of heterogeneity, horizontal pleiotropy, and outliers (Table 2), and the “leave-one-out” analysis showed that the MR results were not affected by a single SNP (Figure 5C).

The random effects IVW results showed that X-11317 had a negative genetic causal relationship with SpA (Supplementary Figure S3A,B). The Cochran’s Q statistic (MR-IVW) and Rucker’s Q statistic (MR Egger) showed that there was no heterogeneity (*p* > 0.05). The intercept test (MR Egger) and global test (MR-PRESSO) showed that there was no horizontal pleiotropy (*p* > 0.05). The distortion test (MR-PRESSO) showed that there were no outliers (Supplementary Table S2). However, the “leave-one-out” analysis showed that the MR results were affected by four SNPs (rs7837038, rs6593265, rs7499892, and rs11832861) (Supplementary Figure S3B). The second round of MR analysis was performed after rs7837038, rs6593265, rs7499892, and rs11832861 were removed. The random effects IVW results showed that X-11317 had no genetic causal relationship with SpA (*p* = 0.667, OR = 0.998). The MR Egger, weighted median, simple mode, and weighted mode were consistent with the random effects IVW results (Figure 2 and Figure 4D). There were no instances of heterogeneity, horizontal pleiotropy, and outliers (Table 2), and the “leave-one-out” analysis showed that the MR results were not affected by a single SNP (Figure 5D).

The random effects IVW results showed that X-12510 had no genetic causal relationship with SpA (Supplementary Figure S4A,B). The Cochran’s Q statistic (MR-IVW) and Rucker’s Q statistic (MR Egger) showed that there was no heterogeneity (*p* > 0.05). The intercept test (MR Egger) and global test (MR-PRESSO) showed that there was no horizontal pleiotropy (*p* > 0.05). The distortion test (MR-PRESSO) showed that there were no outliers (Supplementary Table S2). The “leave-one-out” analysis showed that the MR results were affected by one SNP (rs13538) (Supplementary Figure S4B). Therefore, the second round of MR analysis was performed after rs13538 was removed. The random effects IVW results showed that X-12510 had a positive genetic causal relationship with SpA (Supplementary Figure S4C,D), and there were no instances of heterogeneity, horizontal pleiotropy, and outliers (Supplementary Table S2), but the “leave-one-out” analysis showed that the MR results were affected by two SNPs (rs1165209 and rs4865204) (Supplementary Figure S4D). Finally, the third round of MR analysis was performed after rs1165209 and rs4865204 were removed. The random effects IVW results showed that X-12510 had no genetic causal relationship with SpA (*p* = 0.446, OR = 1.003). The MR Egger, weighted median, simple mode, and weighted mode were consistent with the random effects IVW results (Figure 2 and Figure 4E). There were no instances of heterogeneity, horizontal pleiotropy, and outliers (Table 2), and the “leave-one-out” analysis showed that the MR results were not affected by a single SNP (Figure 5E).

The random effects IVW results showed that X-13859 had no genetic causal relationship with SpA (Supplementary Figure S5A,B). The Cochran’s Q statistic (MR-IVW) and Rucker’s Q statistic (MR Egger) showed that there was no heterogeneity (*p* > 0.05). The intercept test (MR Egger) showed that there was no horizontal pleiotropy (*p* > 0.05), but the global test (MR-PRESSO) showed that there was horizontal pleiotropy (*p* < 0.05). The distortion test (MR-PRESSO) showed that there was one outlier (rs2683822) and two potential outliers (rs10763882 and rs12570090) (Supplementary Table S2). The “leave-one-out” analysis showed that the MR results were not affected by a single SNP (Supplementary Figure S5B). In order to remove horizontal pleiotropy, we performed a second round of MR analysis after removing the one outlier. The random effects IVW results showed that X-13859 had no genetic causal relationship with SpA (*p* = 0.852, OR = 0.999). The MR Egger, weighted median, simple mode, and weighted mode were consistent with the random effects IVW results (Figure 2 and Figure 4F). There were no instances of heterogeneity, horizontal pleiotropy, and outliers (Table 2), and the “leave-one-out” analysis showed that the MR results were not affected by a single SNP (Figure 5F).

## 4. Discussion

Although there had studies on SpA and blood metabolites, the link between SpA and blood metabolites is not yet understood. We are the first to systematically study the genetic correlation and causal relationship between SpA and blood metabolites. LDSC regression found seven blood metabolites genetically related to SpA, including X-10395, pantothenate, caprylate, pelargonate, X-11317, X-12510, and X-13859. Furthermore, MR analysis found that X-10395 had a positive genetic causal relationship with SpA. Our research is different from traditional observational research, as it is based on the analysis of genotype data. GWAS summary data have a large sample size, the LDSC regression has a strong ability to estimate genetic correlation, and MR analysis can effectively evaluate genetic causality, so our results are reliable. Our results have great potential for furthering the understanding of the genetic mechanism of SpA and developing effective diagnostic indicators.

Pantothenate (vitamin B5) plays an important role in mammalian cell metabolism. Pantothenate is not only an essential vitamin for coenzyme A (CoA) biosynthesis in mammalian cells but is also an integral part of acyl carrier proteins (ACPs), fatty acid synthetase complexes, and fatty acid synthesis [41]. Pantothenate is also related to inflammation. Pantothenate regulates the proportion of macrophages, promotes the expression of interferon-γ and IL-17 in CD^4+^ T cells, and can regulate immunity by increasing the production of inflammatory cytokines in epithelial cells [42]. One study found that in men and women aged 40 or over in South Korea, the intake of pantothenate is inversely proportional to the subsequent C-reactive protein (CRP) concentration [43]. Vitamin D deficiency changes the gut microbiota and reduces the production of vitamin B in the intestine. The resulting lack of pantothenate will produce a proinflammatory state related to atherosclerosis and autoimmunity [44]. Vitamin B complex (B1, B2, B3, B5, B6, and B12) treatment can reduce local inflammation after peripheral nerve injury, not only reducing the expression of proinflammatory cytokines but also increasing the expression of anti-inflammatory cytokines [45]. From this, we can infer that pantothenate levels are inversely proportional to inflammatory cytokine activity and that pantothenate can reduce inflammation. A previous study on pantothenate and osteoarthritis (OA) also used anti-lactate calcium tablets containing calcium D-antothenate and L-cysteine hydrochloride in the treatment of knee OA with a placebo, but it was found that the improvement of symptoms in the patients taking pantothenate was a placebo response [46]. It has also been found that acute pantothenate deficiency can lead to pathological changes in the joints in rats that are very similar to the pathological changes in OA, osteoporosis, cartilage calcification, osteophytes, and cleft lip formation [47]. The pantothenate level in the whole blood of patients with RA is significantly lower than the normal level, and the severity of signs and symptoms is inversely proportional to the pantothenate blood concentration [47]. Oral pantothenate supplements can reduce symptoms in patients with OA and RA [47]. Pantothenate has a dual role in osteoclast differentiation. Low-concentration pantothenate induces osteoclast differentiation by stimulating the PI3K-Akt pathway, while high-concentration pantothenate inhibits the formation of osteoclasts by scavenging reactive oxygen species [48]. Osteoclast differentiation and dysfunction can cause osteoporosis, osteopathic atrophy, Paget’s disease, and other bone diseases. Pantothenate not only has a potential role in protecting against bone loss and maintaining estrogen content but also has a potential therapeutic role in preventing osteoclast-related diseases [48]. Although the concentration of pantothenate acid in patients with OA and RA is low, its specific role in the pathogenesis of OA and RA is still unclear. We discovered for the first time that pantothenate has a significant genetic correlation with SpA. As SpA is an autoinflammatory disease, the genetic correlation between SpA and pantothenate is likely to be related to the relationship between pantothenate and inflammation [49,50].

Caprylate is a naturally occurring nontoxic long-chain saturated fatty acid present in human breast milk, cow’s milk, and coconut oil and has been used as a traditional protein stabilizer for serum albumin. It contains a hydrophobic alkyl chain and a hydrophilic carboxylate, which acts as a surfactant. The TLR4/NF-κB signaling pathway plays an important role in the stress response and inflammatory response. Toll-like receptor 4 (TLR4) is expressed in vascular endothelial cells and the macrophages of smooth muscle. It is triggered by the TLR4 ligand to activate the NF-κB signaling pathway and increase the gene transcription of many proinflammatory factors [51]. MicroRNA 93 inhibits chondrocyte apoptosis and inflammation in OA by targeting the TLR4/NF-κB signaling pathway [52]. Caprylate inhibits inflammation through the TLR4/NF-κB signaling pathway, thereby improving atherosclerosis in ApoE-deficient mice [53]. Studies have found that the NF-κB signaling pathway is also required for the development of AS. TLR4/NF-κB signaling pathways is very important in the regulation of the AS inflammatory response. Tumor necrosis factor-α (TNF-α) inhibitors inhibit the inflammatory response in AS by increasing the expression of TLR3 and inhibiting TLR4 and TLR5 expression and the NF-κB signaling pathway [54]. Caprylate can inhibit inflammation through the TLR4/NF-κB signaling pathway. Caprylate may also be involved in the regulation of the NF-κB signaling pathway in the AS inflammatory response. We found for the first time an obvious genetic correlation between caprylate and SpA. We speculate that caprylate may inhibit the inflammatory response involved in SpA through the TLR4/NF-κB signaling pathway. Further study of the specific mechanism will be of great value for understanding the genetic mechanism of SpA and for improving the diagnostic and treatment criteria.

Pelargonate is soluble in organic solvents such as ethanol, chloroform, and ether but insoluble in water. The natural product (free or esterified state) of pelargonate is found in essential oils such as rose, geraniol, and lavender. It can also be organically synthesized from oleic acid by nitric acid or ozone oxidation. Heptanoate, pelargonate, and caprylate levels in the visceral fat (not seen in subcutaneous fat) are significantly higher in postmenopausal women. Fatty acid metabolism changes in visceral adipose tissue may be related to metabolic syndrome [55]. However, chronic inflammation of visceral adipose tissue is causally related to metabolic syndrome, and pentadecanoic acid and palmitate can induce proinflammatory responses in macrophages [55,56]. We found that both pelargonate and caprylate have obvious genetic correlations with SpA, and fatty acid metabolism changes in visceral adipose tissue in postmenopausal women are related to the causal relationship between chronic inflammation of visceral adipose tissue and metabolic syndrome. Therefore, we speculate that chronic inflammation in the visceral adipose tissue may bridge the genetic correlation between nonanoic acid, octanoic acid, and SpA, but further studies are needed to verify their relationship.

Although pantothenate, caprylate, and pelargonate are not directly related to SpA, they are all related to inflammation, which also reflects the reliability of our research results. In addition, we also found four unnamed blood metabolites (X-10395, X-11317, X-12510, and X-13859) that have a significant genetic correlation with SpA. Moreover, there is a positive causal relationship between X-10395 and SpA. X-10395 may be the risk factor of SpA, and it provides a new research direction for investigating the diagnostic indicators of SpA. None of the four unnamed blood metabolites (X-10395, X-11317, X-12510, and X-13859) have been definitively reported, and we do not know much about them. However, this does not negate the results of our study. On the contrary, the four unnamed blood metabolites found in this study provide a new research direction for further research on SpA.

Our research also has some limitations. First, all subjects in this study were of European ancestry. Our findings may need to be interpreted with caution when applied to other groups due to differences in genetic background. The ethnicity and selection bias of the samples influence causality, so further research on other populations is needed, and further large-sample studies and biological investigations are needed to confirm our findings. Second, genetic polymorphism can usually only explain a small part of the total variance of traits, and causal inference based on simple IV studies has certain limitations. Therefore, more studies are needed to verify the results of our MR analysis. Finally, four of the blood metabolites we identified have not yet been named, and their specific association with SpA cannot be further researched at this time.

## 5. Conclusions

For the first time, we used the GWAS summary data to analyze the genetic correlation and causality between the SpA and blood metabolites. X-10395, pantothenate, caprylate, pelargonate, X-11317, X-12510, and X-13859 have genetic correlations with SpA, and there is a positive causal relationship between X-10395 and SpA. We explored the relationship between SpA and human blood metabolites from a genetic perspective. No similar results have been reported, and our study provides a new direction for the investigation of the genetic mechanisms of SpA and its diagnostic indicators. We will further study the specific genetic and causal relationship between these seven blood metabolites and SpA to further understand the genetic mechanisms and diagnostic indicators of SpA.

## Figures and Tables

**Table 1:** Genetic correlation between human blood metabolites and SpA.

Blood Metabolites		Correlation Coefficient	*p*-Values
X-10395	SpA	−0.546	0.025
Pantothenate		−0.565	0.038
Caprylate		−0.333	0.037
Pelargonate		−0.339	0.047
X-11317		−0.350	0.038
X-12510		−0.399	0.034
X-13859		−0.458	0.015

**Table 2:** Sensitivity analysis of the MR analysis results of human blood metabolites and spondyloarthritis.

Exposure	Outcome	Heterogeneity Test		Pleiotropy Test	MR-PRESSO	
		Cochran’s Q Test (*p*-Value)	Rucker’s Q Test (*p*-Value)	Egger Intercept (*p*-Value)	Distortion Test	Global Test
		IVW	MR-Egger	MR-Egger	Outliers	*p*-Value
X-10395	SpA	0.733	0.858	0.115	0	0.741
Pantothenate		0.802	0.804	0.388	0	0.251
Caprylate		0.753	0.731	0.498	0	0.798
Pelargonate		0.578	0.641	0.213	0	0.670
X-11317		0.674	0.619	0.783	0	0.404
X-12510		0.639	0.623	0.428	0	0.659
X-13859		0.264	0.352	0.113	0	0.084

## Data Availability

The data used in this article come from UK Biobank (http://geneatlas.roslin.ed.ac.uk/).

## Supplementary Materials

https://www.mdpi.com/article/10.3390/jcm12031201/s1, Figure S1: MR analysis process of the X-10395 and spondyloarthritis; Figure S2: MR analysis process of the pelargonate and spondyloarthritis; Figure S3: MR analysis process of the X-11317 and spondyloarthritis; Figure S4: MR analysis process of the X-12510 and spondyloarthritis; Figure S5: MR analysis process of the X-13859 and spondyloarthritis; Table S1: The instrumental variable of the MR analysis; Table S2: MR analysis process of the human blood metabolites and spondyloarthritis.
