# Supplementary material for: Large-Scale Genetic Correlation Analysis between Spondyloarthritis and Human Blood Metabolites
Source: J Clin Med. 2023 Feb 2;12(3):1201. doi: 10.3390/jcm12031201 (PMC9917834; doi:10.3390/jcm12031201)
Supplement: Supplementary file 1 [file jcm-12-01201-s001.zip › Supplementary Figure S1-S5.pdf]

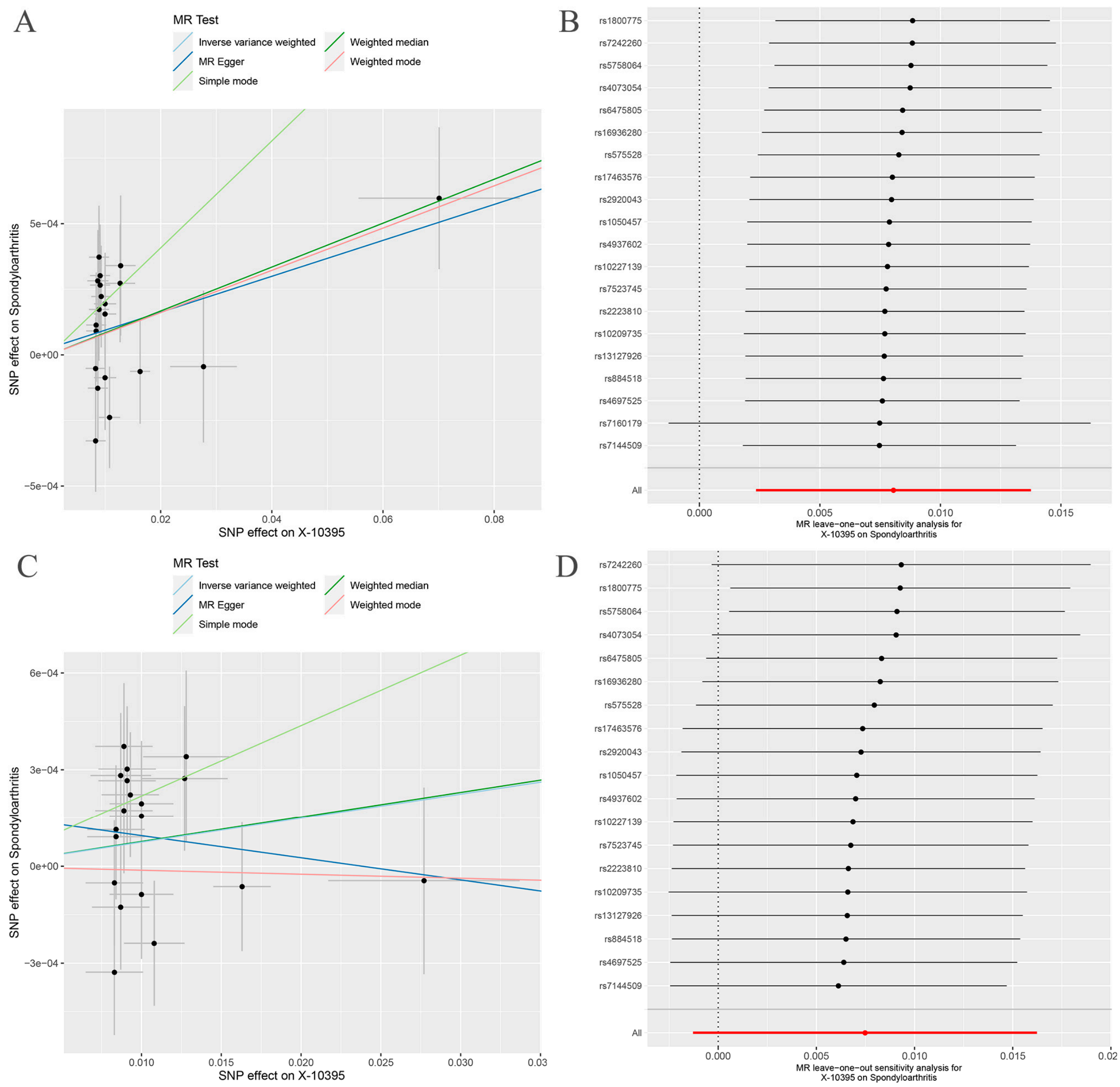

Figure S1: MR analysis process of the X-10395 and spondyloarthritis.

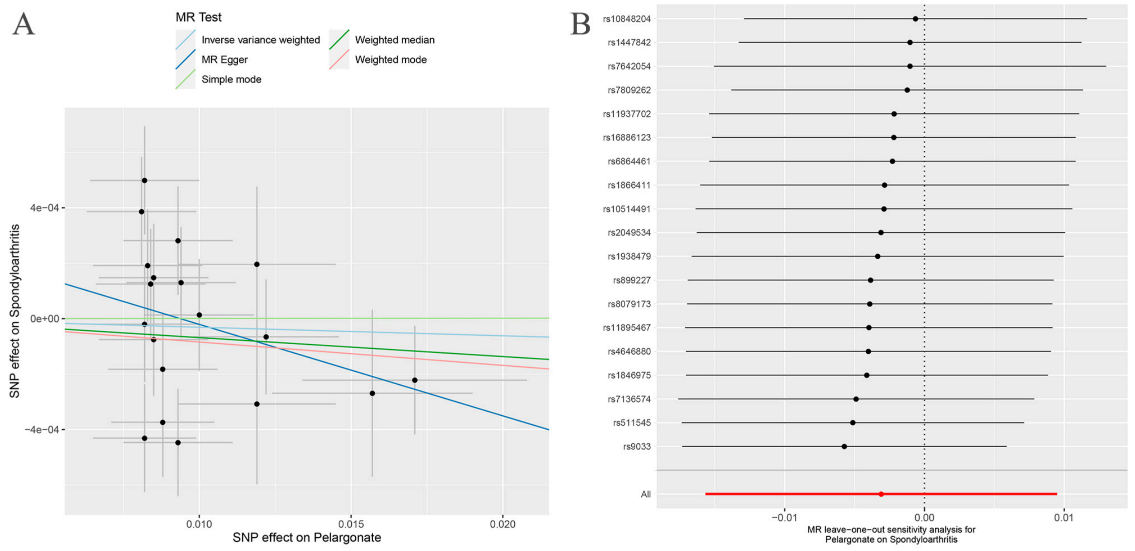

Figure S2: MR analysis process of the pelargonate and spondyloarthritis.

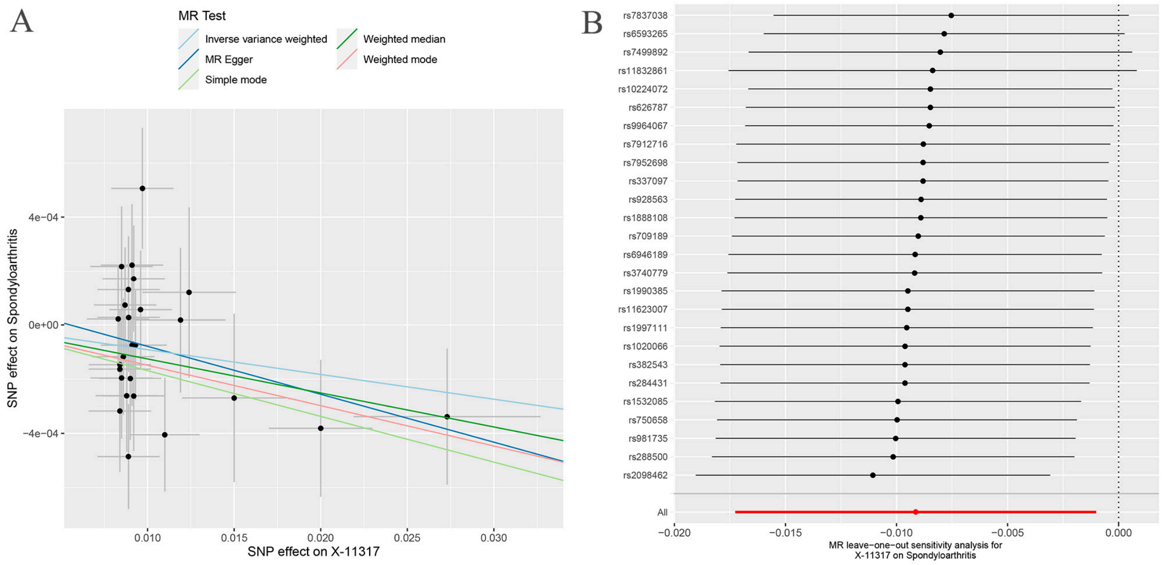

Figure S3: MR analysis process of the X-11317 and spondyloarthritis.

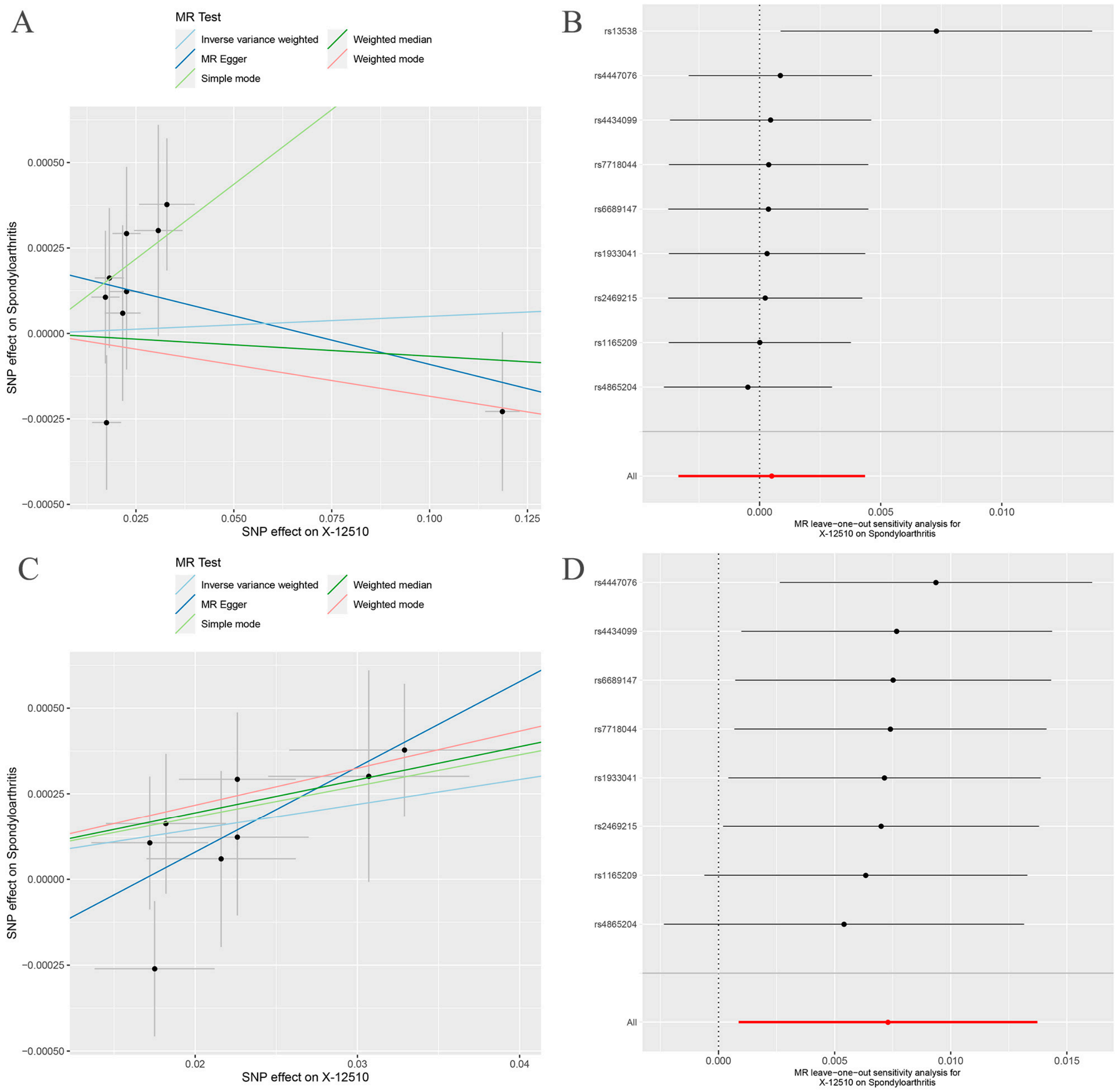

Figure S4: MR analysis process of the X-12510 and spondyloarthritis.

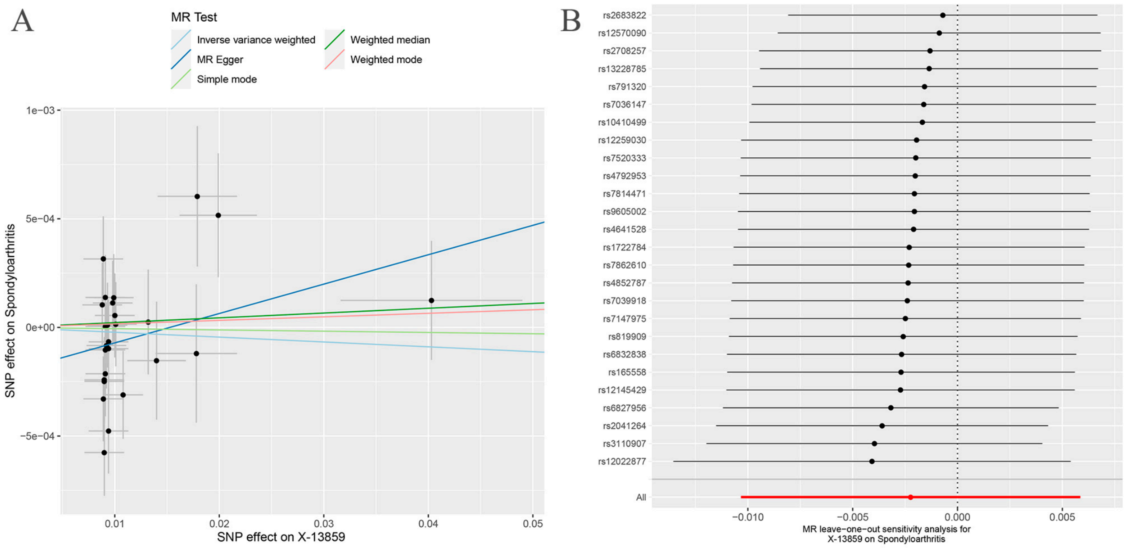

Figure S5: MR analysis process of the X-13859 and spondyloarthritis.
