# Supplementary material for: Large-Scale Genetic Correlation Analysis between Spondyloarthritis and Human Blood Metabolites
Source: J Clin Med. 2023 Feb 2;12(3):1201. doi: 10.3390/jcm12031201 (PMC9917834; doi:10.3390/jcm12031201)
Supplement: Supplementary file 1 [file jcm-12-01201-s001.zip › Supplementary Table S2.pdf]

Supplementary table S2: MR analysis process of the human blood metabolites and spondyloarthritis

**Exposure**	**Outcome**	**Heterogeneity Test**		**Pleiotropy Test**	**MR-PRESSO**			**Leave One Out**
		**Cochran's Q Test (*P* value)**	**Rucker's Q Test (*P* value)**	**Egger Intercept (*P* value)**	**Distortion Test**	**Distortion Test**	**Global Test**	
		**IVW**	**MR-Egger**	**MR-Egger**	**Outliers**	**Potential outliers**	***P* value**	
X-10395 1	SpA	0.421	0.365	0.725	0	0	0.514	1
X-10395 2		0.359	0.371	0.296	0	0	0.374	2
Pelargonate 1		0.017	0.023	0.262	0	4	0.034	0
X-11317 1		0.370	0.348	0.462	0	0	0.260	4
X-12510 1		0.206	0.365	0.127	0	0	0.347	1
X-12510 2		0.648	0.770	0.229	0	0	0.674	2
X-13859 1		0.074	0.145	0.074	1	2	0.023	0
