# Supplementary figures and images for: Large-Scale Genetic Correlation Analysis between Spondyloarthritis and Human Blood Metabolites
Source: J Clin Med. 2023 Feb 2;12(3):1201. doi: 10.3390/jcm12031201 (PMC9917834; doi:10.3390/jcm12031201)

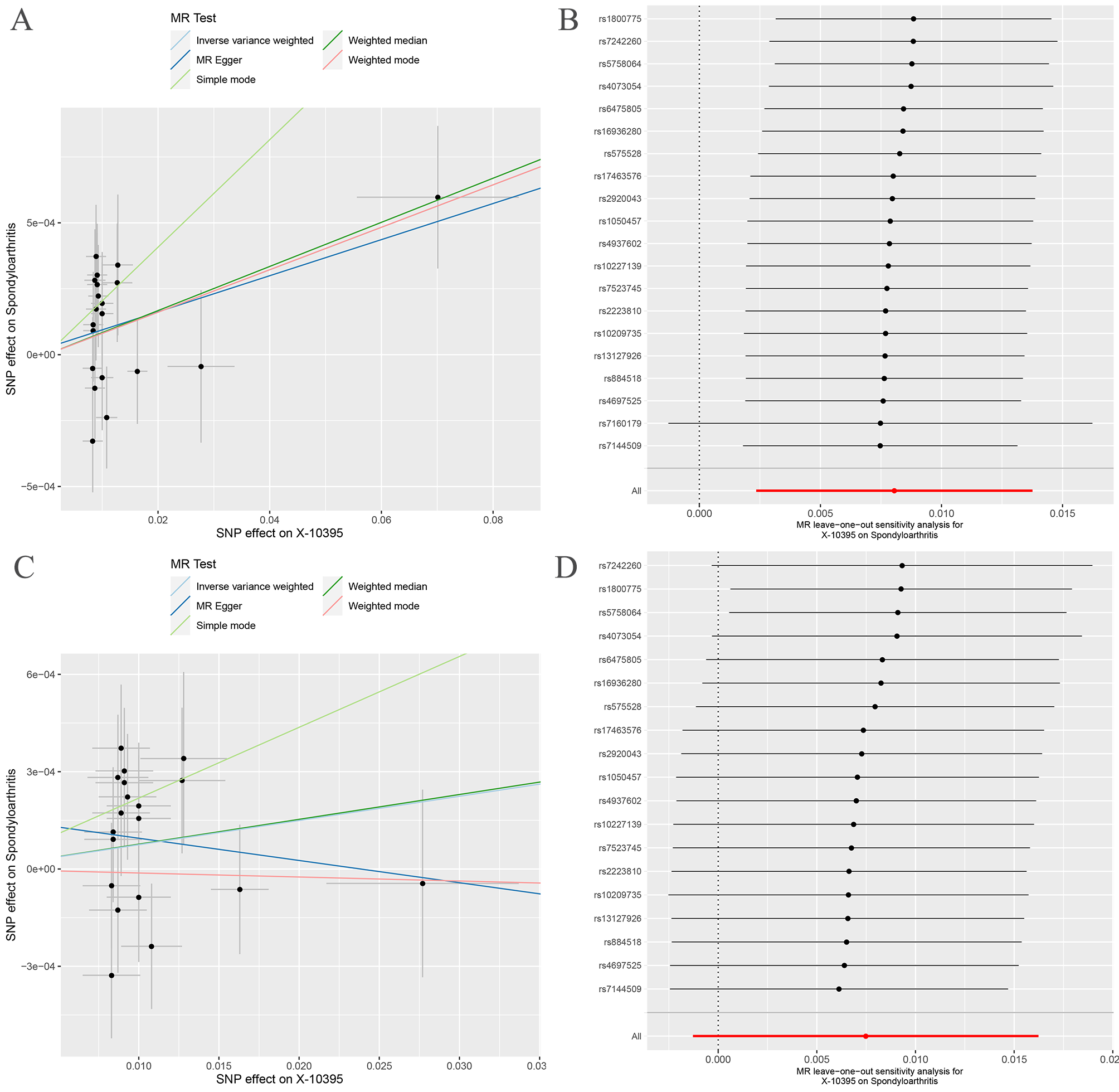

Supplement: Supplementary file 1 [file jcm-12-01201-s001.zip › Supplementary Figure S1.tif]

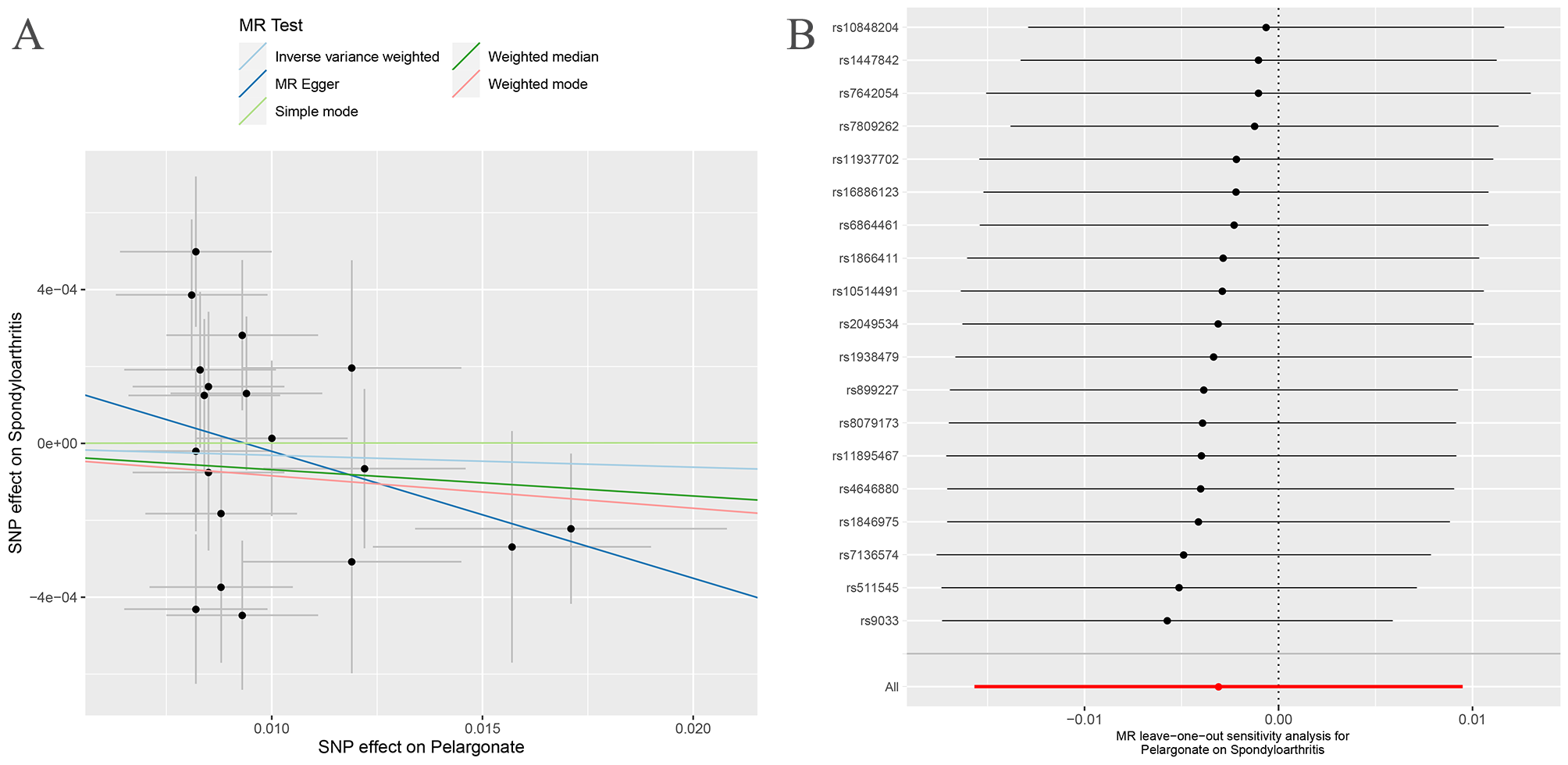

Supplement: Supplementary file 1 [file jcm-12-01201-s001.zip › Supplementary Figure S2.tif]

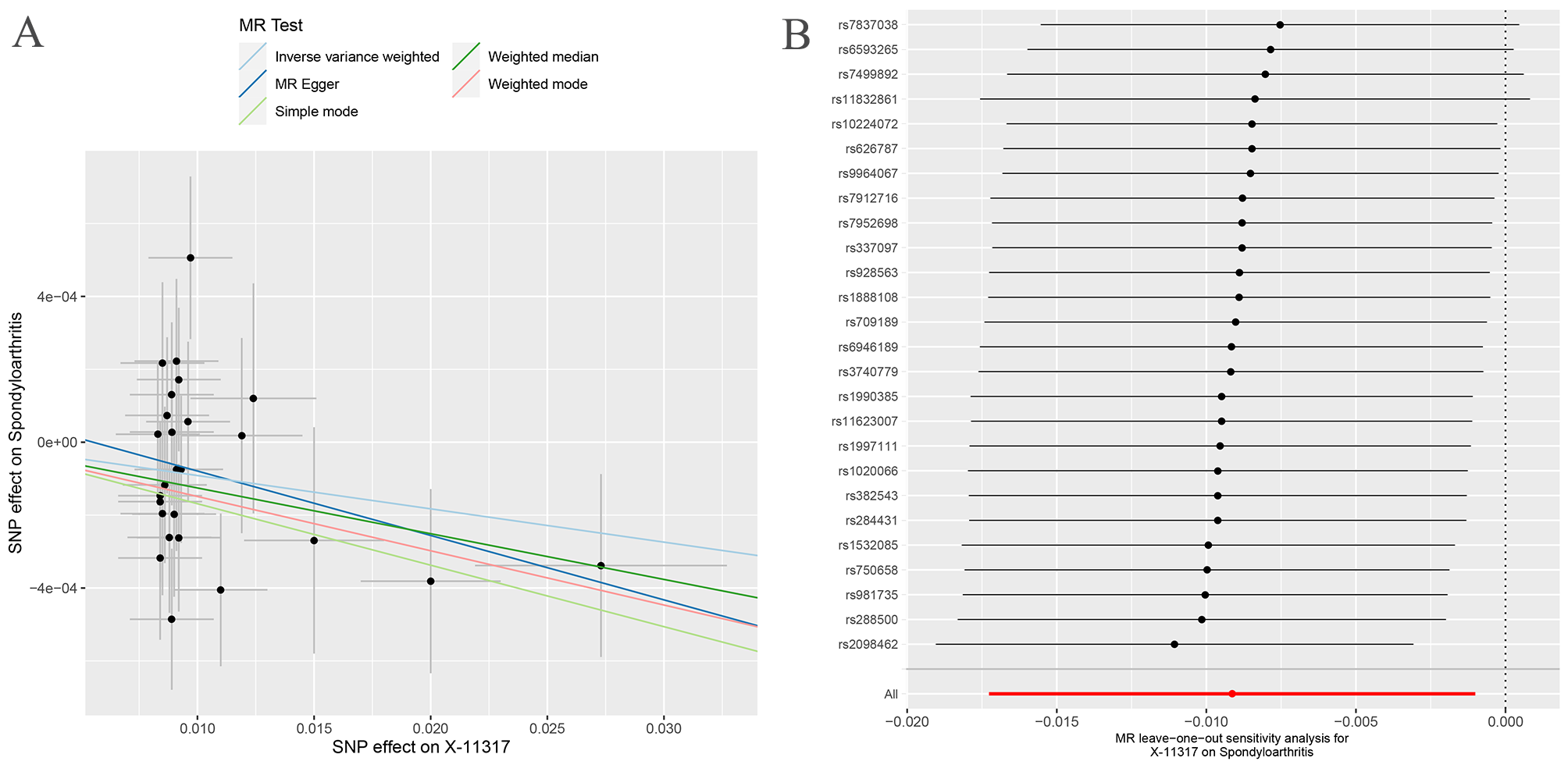

Supplement: Supplementary file 1 [file jcm-12-01201-s001.zip › Supplementary Figure S3.tif]

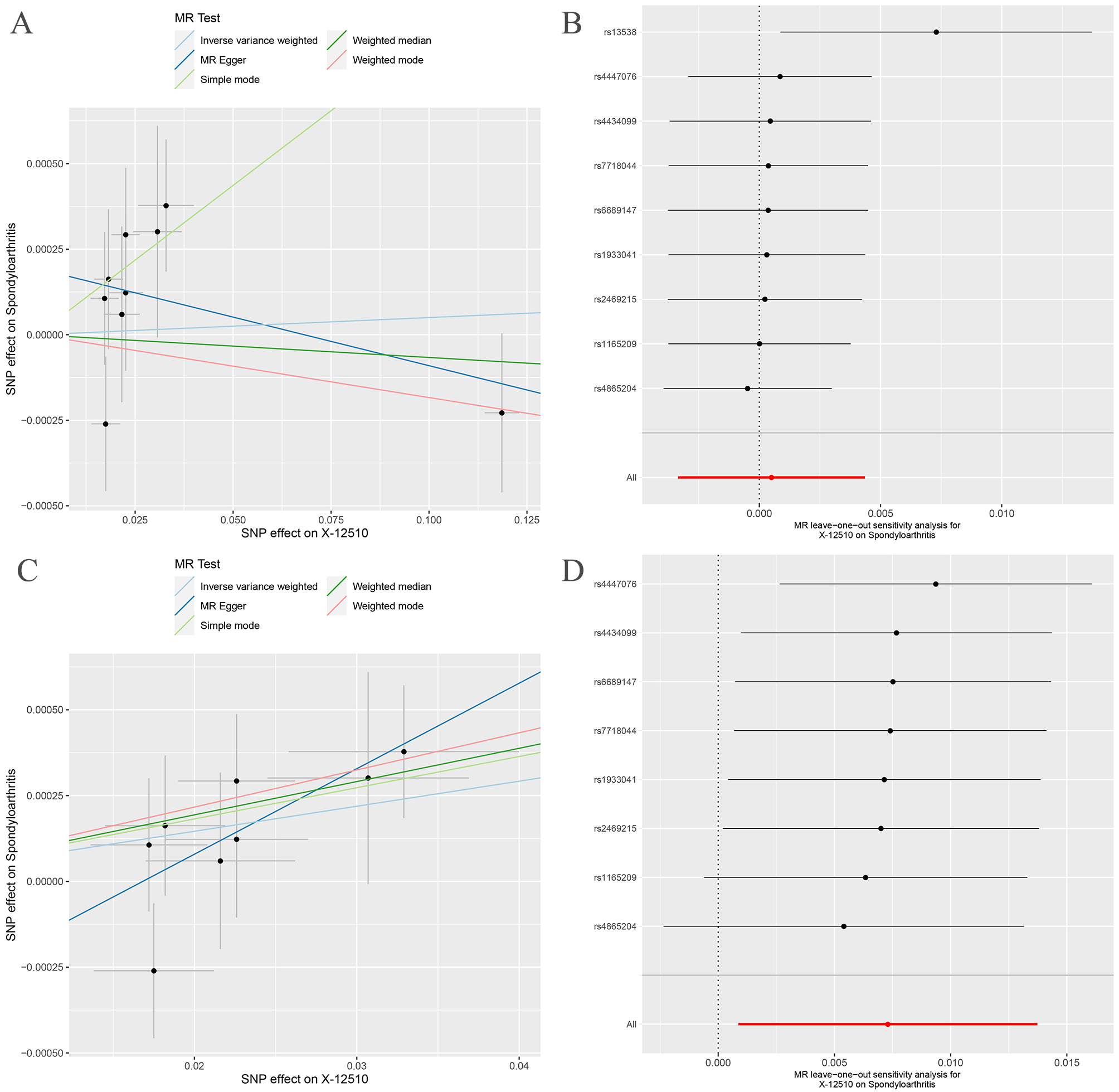

Supplement: Supplementary file 1 [file jcm-12-01201-s001.zip › Supplementary Figure S4.tif]

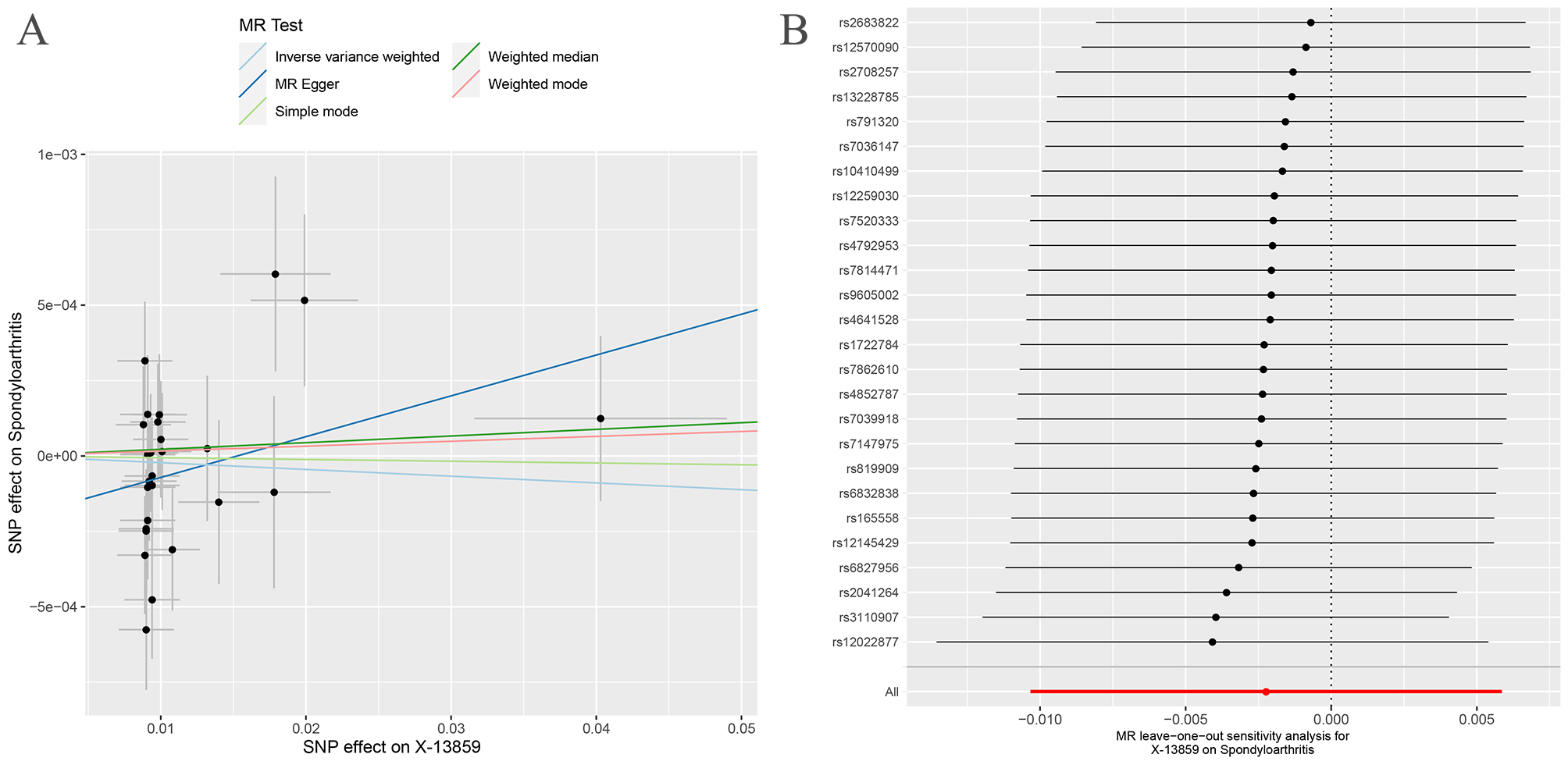

Supplement: Supplementary file 1 [file jcm-12-01201-s001.zip › Supplementary Figure S5.tif]
